# Genetic Test for Dilated and Hypertrophic Cardiomyopathies: Useful or Less Than Useful for Patients?

**Published:** 2013-01-04

**Authors:** F Pastore, V Parisi, R Romano, G Rengo, G Pagano, K Komici, D Leosco

**Affiliations:** 1 Department of Cardiology, AOU “Maggiore Della Carità”, Novara; 2 Department of Clinical Medicine, Cardiovascular and Immunological Sciences, University of Naples “Federico II”; 3 Department of Medicine, University of Salerno

**Keywords:** Dilated Cardiomyopathy, Hypertrophic Cardiomyopathy, Genetic Testing

## Abstract

Genetic testing for potentially heritable cardiomyopathies has advanced from basic scientific discovery to clinical application. Nowadays, genetic diagnostic tests for cardiomyopathies are clinically available. As a consequence is fundamental the understanding of the clinical utility, in terms of diagnosis and prognosis, of genetic test results. In addition, the genetic counselling, regarding risks, benefits and options, is recommended for all patients and their relatives.

However the relation between genotype and phenotype remains often unclear, and there is frequently a variance of uncertain significance. Consequently, the genetic test should always be approached as one component of a comprehensive cardio-genetic evaluation.

This review aims to explore when genetic tests are indicated in patients with dilated and hypertrophic cardiomyopathy.

## I. INTRODUCTION

The first gene-mutation causing cardiomyopathy was discovered more than thirty years ago. Nowadays the genic-mutations are at least 500 just for Hypertrophic Cardiomyopathy. However, the clinical beneficial of a genetic-test result remains often unclear and questionable. Usually, the first approach to a patient with a cardiomyopathy is guided by the phenotype. Anamnesis and instrumental investigations, and above all electrocardiography and echocardiography, are extremely useful. Another diagnostic investigation, performed in specialized centres, is magnetic resonance imaging (MRI). It is very important to characterize the muscle tissue and the interstitial space. A detailed family history could guide the diagnosis of a familial genetic disease, although there is the possibility of a *de novo* mutation that should be suspected in the absence of a positive family history for disease. Different mutations in the same gene might cause different disease phenotype and different disease severity. For example laminin A/C mutations might cause isolated Dilated Cardiomyopathy (DCM), Emery-Dreifuss Muscular Dystrophy, or disorders without DCM. Cardiologists should know the feasibility of a genetic diagnosis, its clinical relevance and its potential impact on prognosis. Another fundamental aspect is the genetic counselling, finalized to discuss the significance of the identification of the genetic mutation with the patient and its relevance in his/her life. Contrary to common misperception, genetic tests are probabilistic and not deterministic tests. Many positive tests are represented by DNA variants of uncertain clinical significance. The genetic testing for cardiomyopathies has some ethical problems: is it right to consider sick a patient with a mutation indicative of disease if he might never show the phenotype?

## II. DILATATED CARDIOMYOPATHY

Dilated Cardiomyopathy is characterized by the systolic dysfunction and the left ventricular (LV) dilatation with the progressive LV failure. Although this disease has various etiopathogenesis, the term cardiomyopathy refers to genetic cardiomyopathy. Baig et al showed that the patient with DCM have a familiar DCM for 48% of cases, when asymptomatic LV dysfunction was considered the first sign on DCM [1–3]. The familiar screening is strongly recommended in familiar of patients with DCM [4]. However the role of genetic test is unclear.

*Genetic test for dilated Cardiomyopathy.* More than 30 genes have been identified as cause of DCM showing a marked locus heterogeneity. The genes implicated encode proteins involved in the structure of the cardiomyocyte as cytoskeletal proteins, myofilament proteins and ion channels. Mitochondrial defects have also been identified(Table 1)[5]. This heterogeneity highlights the various mechanisms involved in DCM. This disease is likely final phenotype of reduced contractile force of cardiomyocytes. Interestingly, some mutations of the genes causing DCM can cause also hypertrophic cardiomyopathy (HCM), clarifying the importance of secondary factors as modifiers genes and environment to determine the phenotype. Most genetic DCM inheritance follows an autosomal dominant pattern, although X-linked, recessive, and mitochondrial patterns of inheritance occur. The sensitivity of genetic is estimated at 20% and none of genes appears to account for 5% of familial DCM. This low sensitivity is very important and it underlines the little diagnostic power of genetic testing in not-selected people. The sensitivity is higher in specific forms as the DCMs associated with conduction defects. Genetic DCM shows age-dependent penetrance and a variable expression. The same mutation can result in a different phenotype in members of the same family, underlying the importance of others factors [6]. Some mutations are more aggressive and they can often cause sudden death, e.g. mutations of laminin and desmin [7].

When the mutation of the proband is known, the family genetic screening is very helpful to diagnose eventual early DCM. However clinicians should always keep in mind that, considered the heterogeneity of expression, some carriers would never develop the disease. The family screening is mandatory for the aggressive gene-mutations as mutations of laminin. Indeed the gene testing changes prognosis only for laminin mutations. In conclusion, clinically the genetic testing is recommended only for family screening and especially for aggressive mutations (table 2). However, it is very important to evaluate the mutations for research purposes, especially for possible gene therapy in future.

## III. HYPERTROPHIC CARDIOMYOPATHY

Hypertrophic cardiomyopathy (HCM) is a common disease, that affects 1 in 500 people [8]. Usually it is inherited as an autosomal dominant trait, instead *de novo* mutations are rare [9]. The phenotype of a patient with HCM is characterized by asymmetrical cardiac hypertrophy, that doesn’t have any evident cause, myocyte disarray, and fibrosis. Patients show a marked phenotypic variability, even within the same family, and an incomplete penetrance [8]. HCM is one of the most frequently identified causes of sudden cardiac death caused by the high prevalence of malignant arrhythmias. Although many patients with HCM are asymptomatic and sudden death may be unpredictable, genetic screening in families is essential for prevention. Mutations in MYH7 and MYBPC3 genes, that encod for the beta-myosin heavy chain and the cardiac myosin binding protein-C, are present in about 80% of HCM cases [10,11]. Mutations in other genes, such as TNNT2, TNNI3, and TPM1, encod for proteins of the troponin complex and occur in 10% to 15% of HCM patients [12]. 9 genes are used for genetic testing. Considering all genetic testing these 9 genes, a mutation is identified in 40% to 60% of sporadic and familial cases[10]. Anyway, the relation between genotype and phenotype remains elusive, because of extreme genetic heterogeneity, variation in penetrance and expressivity, even considering individuals carrying identical mutations. Cases with a negative HCM genetic test might have HCM-causing mutations in unexplored regions within the known HCM genes or in undiscovered genes.

Rarely the phenotype is related to underlying HCM disease gene, and so it might bepoorlyuseful for managing patients. Mutations in MYH7 alleles usually are associated with an important clinical disease expression. Instead, mutations in MYBPC3 alleles have been associated with later onset disease [13]. Patients with TNNT2 mutations usually show a lower severity of LV hypertrophy but a higher arrhythmia risk [14]. Only few specific mutations might carry a prognostic implication. That is the reason why a genetic test result in isolation will not constitute an indication for an ICD for primary prevention.

In conclusion, genetic testing is recommended for patients with a clinical diagnosis of HCM when the genetic testing benefit family members and potentially other relatives (table 3). It is recommended in families with a history of sudden death, in families in which numerous relatives are at risk and that need periodic clinical evaluation without the genetic testing, and when the clinical diagnosis is difficult. It might be recommended even thegenetic analysis of post mortem samples if there is a case of sudden death in a family where HCM was not previously known. Family screening is recommended for all first-degree relatives and it is important also for cost-effectiveness reasons. Indeed, when a family member has anegative genetic testing result, he/she can be discharged and there is no reason for clinical investigations or long-term follow-up [15]. Long-term studies are required to accumulate reliable evidence on genotype–phenotype relations.

## Figures and Tables

**Table 1.** **Dilated Cardiomyopathy (DCM): genes mutated in DCM.** The most common genes mutated in DCM and the proteins that they codified for. Modified from: Hershberger RE, Morales A, Siegfried JD. Clinical and Genetic Issues in Dilated Cardiomyopathy: a Review for Genetics Professionals. Genet Med 2010; 12(11): 655–67.

**Gene**	**Protein**	**Function**
*ACTC*	cardiac actin	Sarcomeric protein; muscle contraction
*DES*	desmin	DAGC; transduces contractile forces
*SGCD*	δ-sarcoglycan	DAGC; transduces contractile forces
*MYH7*	β-myosin heavy chain	Sarcomeric protein; muscle contraction
*TNNT2*	cardiac troponin T	Sarcomeric protein; muscle contraction
*TPM1*	α-tropomyosin	Sarcomeric protein; muscle contraction
*TTN*	titin	Sarcomere structure/extensible scaffold for proteins
*VCL*	metavinculin	Sarcomere structure; intercalated discs
*MYBPC3*	myosin-binding protein C	Sarcomeric protein; muscle contraction
*MLP/CSRP3*	muscle LIM protein	Sarcomere stretch sensor/ Z discs
*ACTN2*	α-actinin-2	Sarcomere structure; anchor for myofibrillar actin
*PLN*	phospholamban	Sarcoplasmic reticulum Ca++ regulator; inhibits SERCA2 pump
*ZASP/LDB3*	Cypher	Cytoskeletal assembly; targeting/clustering of membrane proteins
*MYH6*	α-myosin heavy chain	Sarcomeric protein; muscle contraction
*ABCC9*	SUR2A	Kir6.2 regulatory subunit, inwardly rectifying cardiac KATP channel
*TNNC1*	cardiac troponin C	Sarcomeric protein; muscle contraction
*titin-cap TCAP*	titin-cap or telethonin	Z-disc protein that associates with titin; aids sarcomere assembly
*TNNI3*	cardiac troponin I	sarcomeric protein, muscle contraction; also seen as recessive
*EYA4*	eyes-absent 4	Transcriptional coactivators (Six and Dach)
*TMPO*	thymopoietin	Also LAP2; a lamin-associated nuclear protein
*PSEN1/2*	presenilin 1 / 2	Transmembrane proteins, gamma secretase activity
*CRYAB*	alpha B crystalin	Cytoskeletal protein
*PDLIM3*	PDZ LIM domain protein 3	Cytoskeletal protein
*MYPN*	myopalladin	Sarcomeric protein, z-disc
*LAMA4*	laminin a-4	Extracellular matrix protein
*ILK*	integrin-linked kinase	Intracellular ser-threo kinase; interacts with integrins
*RBM20*	RNA binding protein 20	RNA binding protein of the spliceosome
*LMNA*	lamin A/C	Structure/stability of inner nuclear membrane
*SCN5A*	sodium channel	Controls sodium ion flux
*DMD*	dystrophin	DAGC; transduces contractile force

**Table 2:** **Recommendations to genetic testing in Dilated Cardiomyopathy.** Modified from HRS/EHRA Expert Consensus Statement on the State of Genetic Testing for the Channelopathies and Cardiomyopathies [16]

**STATE OF GENETIC TESTING FOR DILATED CARDIOMYOPATHY (DCM)**	
*Class I*	
✓	Patients with a clinical diagnosis of DCM and significant cardiac conduction disease and/or a family history of premature unexpected sudden death have been recommended for a comprehensive or targeted (LMNA and SCN5A) DCM genetic testing.
✓	Family members and appropriate relatives of an index case have been recommended for a mutation-specific genetic testing

*Class IIa*	
✓	Patients with familial DCM have been recommended for genetic testing to confirm the diagnosis, to recognize those who are at highest risk of arrhythmia and syndromic features, to facilitate cascade screening within the family, and to help with family planning.

**Table 3:** **Recommendations to genetic testing for Hypertrophic Cardiomyopathy.** *Modified from HRS/EHRA Expert Consensus Statement on the State of Genetic Testing for the Channelopathies and Cardiomyopathies* [16]

**STATE OF GENETIC TESTING FOR HYPERTROPHIC CARDIOMYOPATHY (HCM)**	
*Class I*	
✓	Patients with a clinical diagnosis of HCM based on examination of the patient’s clinical history, family history, and electrocardiographic echocardiographic phenotype have been recommended for a comprehensive or targeted (MYBPC3, MYH7, TNNI3, TNNT2, TPM1) HCM genetic testing
✓	Family members and appropriate relatives of an index case have been recommended for a mutation-specific genetic testing.
